# Management of temporomandibular joint diseases: a rare case report of coexisting calcium pyrophosphate crystal deposition and synovial chondromatosis

**DOI:** 10.1186/s12903-022-02695-0

**Published:** 2022-12-31

**Authors:** Makoto Murahashi, Edward Hosea Ntege, Masaru Higa, Nobuyuki Maruyama, Toshihiro Kawano, Yusuke Shimizu, Hiroyuki Nakamura

**Affiliations:** 1 Department of Oral and Maxillofacial Surgery, Chubu Tokushukai Hospital, 801 Higa, Kitanakagusuku, Nakagami-gun, Okinawa-ken 901-2393 Japan; 2 Department of Oral and Maxillofacial Functional Rehabilitation, Graduate School of Medicine, University of the Ryukyus, 207 Uehara, Nakagami, Nishihara, Okinawa 903-0215 Japan; 3 Department of Plastic and Reconstructive Surgery, Graduate School of Medicine, University of the Ryukyus, 207 Uehara, Nakagami, Nishihara, Okinawa 903-0215 Japan

**Keywords:** Synovial chondromatosis, Calcium pyrophosphate, Pseudogout, Temporomandibular joint, Crystal arthropathy, Inorganic pyrophosphate, ANKH, Case report

## Abstract

**Background:**

The coexistence of calcium pyrophosphate dihydrate crystal deposition (CPP) and synovial chondromatosis (SC) in the temporomandibular joint (TMJ) is rarely reported. CPP disease (CPPD) is complex arthritis synonymous with excessive pyrophosphate production and variable aberrations in mineral and organic phase metabolism of the joint cartilage, leading to local inundated CPP and crystal deposition of partially deciphered predispositions. Meanwhile, SC is a rare benign synovial joint proliferative disease of unclear etiology and has a low risk of malignant transformation. However, SC manifests severe joint disability and dysfunction because of connective tissue metaplasia of the synovial membrane, which forms cartilaginous nodules with or without calcifications or ossifications. These nodules often detach and form intra-articular loose bodies and very rarely within extraarticular spaces.

**Case presentation:**

We report the case of a 61-year-old man to expand the body of literature on these unusual coexisting arthropathies of the TMJ. The patient presented to our hospital in 2020 with complaints of pain in the right TMJ and trismus for over 6 months. Radiographic assessments of the TMJ provided a preoperative provisional diagnosis of SC. However, the histopathology of the open biopsy revealed tumor-like lesions comprising several deposits of rhomboid and rod-shaped crystals that displayed positive birefringence in polarized light, confirming a coexistence of CPPD. A second-stage operation was performed for the complete removal of the loose bodies and chalk-like lesions including synovectomy. No evidence of recurrence was recorded after a follow-up of nearly 1.5 years.

**Conclusions:**

Isolated CPPD and SC of the TMJ are prevalent in the literature however, monoarticular coexistence of these diseases is rare, due to the lack of consistency in the diagnostic criteria in clinical practice. Moreover, optimal treatment depends on several considerations. This report delineated the molecular etiopathology and underscored the need for continued deciphering of the causal mechanisms of coexisting CPPD and SC of the TMJ. In addition, the importance of confirmatory testing for accurate diagnosis, and appropriate management of these diseases were discussed.

## Background

First identified as pseudogout by McCarty et al. in 1962 [1–3], calcium pyrophosphate crystal deposition (CPPD) is a collective term proposed by Ryan and McCarty in 1985 [4] and later acknowledged by the European Alliance of Associations for Rheumatology (EULAR) task force. It comprises all forms of CPP crystal-induced arthropathies [5]. CPPD is characterized by the deposition of CPP crystals in various intra-articular and/or periarticular tissues, resulting in acute noninfectious inflammatory and degenerative chronic arthropathies and cartilaginous calcifications [6, 7]. CPPD commonly involves large joints such as the knee, shoulder, hip, and wrist and rarely affects small joints such as the temporomandibular joint (TMJ) [8]. CPPD in the TMJ, a complex diarthrodial sliding-ginglymoid synovial joint between the glenoid fossa of the temporal bone and the mandibular condyle, was first described in 1976 by Pritzker et al. [9]. Nonetheless, from a general perspective on its etiopathology, the majority of CPPD cases are of non-genetic type, with partially established etiology (primary idiopathic subtype) and various predisposition factors implicated. For instance, advanced age (> 60 years), osteoarthritis, joint trauma, and metabolic diseases such as hyperparathyroidism, hypothyroidism, hypomagnesemia, hyperphosphatemia, and diabetes mellitus are usually proposed as contributing factors [8, 10–12]. CPPD cases of genetic (familial) type are notably rare, autosomal dominant, and mostly associated with early onset, before the age of 55 years [10]. Multiple studies have identified familial CPPD to have genetic linkages to A1 B12 DR3 human leukocyte antigen (HLA) haplotype and chromosomal region 8q, previously dubbed chondrocalcinosis 1 or chondrocalcinosis with early-onset osteoarthritis (CCAL1) type, and chromosomal region 5p5.1 referred to as chondrocalcinosis 2 (CCAL2) type [13–15]. Specific HLA-associated gene mutations and chromosome 8q-linked CCAL1 are still unknown. However, CCAL2 is reportedly mediated by mutations in the progressive ankylosis protein homolog human gene (*ANKH*) [10, 16]. These gain-of-function mutations affect the regulation of intracellular and extracellular inorganic pyrophosphate (PPi) cellular transporter, subsequently increasing the *ANKH* activity. The increased activity leads to the accumulation of PPi in the cartilage, which in combination with calcium results in CPP crystal formation [17]. Furthermore, *ANKH* mutations also contribute, at least in part, to the pathophysiology of the non-genetic CPPD type [16]. The deposited CPP crystals trigger NALP-3 inflammasome, a Nod-like receptor, and caspase-1-containing cytoplasmic multiprotein complex, to assemble, process, and activate proinflammatory cytokines interleukin (IL)-1*β* and IL-18, which eventually cause joint inflammation [18].

CPPD of the TMJ poses diagnostic challenges; a likely justification for its rarity in the literature [19–21]. Although the diagnosis of CPPD is based on described criteria [22], highlighted in Table 1, the identification of parallelepipedic, principally cellular deposits of crystals with absent or weak positive birefringence in the synovial fluid or tissue of the affected joint (criterion IIa) may be challenged by the presence of other birefringent crystals such as calcium oxalate, synthetic steroids, and ethylenediaminetetraacetic acid in the joint fluids and tissues [23, 24].

**Table 1:** Diagnostic criteria for CPP crystal deposition

Criterion	Description
I	Proof of calcium pyrophosphate crystals by x-ray diffraction or chemical analysis
IIa	Corrected polarization microscopy confirms triclinic and monoclinic crystals showing weak positive birefringence
IIb	Typical calcification image on x-ray
IIIa	Acute arthritis
IIIb	Chronic arthritis
IIIb1	Arthritis in areas where primary OA is usually rare
IIIb2	Narrowing of the joint cleft of the radial carpal joint or patellofemoral joint
IIIb3	Subchondral cyst
IIIb4	Severe progressive degeneration with subchondral bony subsidence and fragmentation with intra-articular radiopaque bodies
IIIb5	Variable osteophyte formation
IIIb6	Tendon calcification
IIIb7	Axial skeletal lesions, subchondral cysts of the epiphyseal and sacroiliac joints, intervertebral disc calcification and vacuum phenomena in multiple vertebral bodies, sacroiliac vacuum phenomena

Diagnostic interpretation: (a) I or IIa + IIb = definitive diagnosis; (b) IIa or IIb = probable or most likely diagnosis; (c) IIIa or IIIb = possible or likely diagnosis

Synovial chondromatosis (SC) is a rare benign joint neoplasm that is associated with the metaplastic proliferation of cartilaginous nodules within the synovial membrane that commonly manifests as intraarticular loose bodies. Although very rare, with a prevalence of approximately 1 per 100,000, loose bodies can form within extraarticular spaces, resulting in a condition referred to as tenosynovial chondromatosis [25–27]. SC affects approximately 1.8 per 1 million individuals, the majority of whom are in their third or fifth decade of life, with an unequal sex ratio, i.e., with a preponderance of men over women. In addition, SC commonly affects the knee and hip in nearly 90% of published cases, but rarely affects the TMJ [28]. Although surgical management, the gold standard treatment for SC, is associated with a high success rate, local recurrence rates of 15–20% have been reported, especially in tenosynovial cases [28]. Moreover, malignant transformation to synovial chondrosarcoma can occur particularly in long-standing disease and multiple recurrence cases but is reportedly extremely rare [29]. Similar to CPPD, the etiopathogenesis of SC is unknown. However, several reports have suggested that SC can be either primary, also referred to as Reichel syndrome, primary synovial osteochondromatosis, or Reichel–Jones–Henderson syndrome, or result from (secondary) degenerative intraarticular changes, joint trauma, and inflammatory events such as neuropathic arthritis and osteochondritis dissecans [27, 30–33]. In their recent report, Agaram, et al. [29] well summarized the genetic alterations identified in SC, which most likely play a role in the disease etiopathology. The genetic alterations include the previously identified *Fibronectin 1* (*FN1*)–*Activin receptor 2A* (*ACVR2A*) gene fusions, which were first identified in SC and reported by Totoki et al. [34] and later demonstrated by several separate molecular studies using various techniques [29]. The authors have also identified a novel lysine (K)-specific methyltransferase 2A (*KMT2A)*–BCL6 corepressor (*BCOR)* gene fusion in a rare case of tenosynovial chondromatosis [29].

The clinical presentation of SC that involves three cardinal signs and symptoms was previously described [35]. The signs and symptoms include preauricular pain, swelling, facial asymmetry, crepitation, joint deformity, and dysfunction, as well as the unilateral deviation of the jaw during mouth opening. Radiology is very useful in demonstrating SC’s features for diagnosis and treatment plans [22, 36–38]. The specific radiographic diagnostic criteria for SC include joint space widening, limitation of motion, irregularity of joint surfaces, presence of calcified loose bodies, and sclerosis of the glenoid fossa and mandibular condyle [39]. The histopathological classification of SC has been described [40] and presented in Table 2.

**Table 2:** Histopathological classification of synovial chondromatosis

Phase	Description
Phase 1	Cartilaginous tissue formation takes place (active synovitis) without loose bodies
Phase 2	Presence of both chondrosynovial changes and intraarticular loose bodies (i.e., nodular synovitis with loose bodies)
Phase 3	Completion of cartilaginous ossification and presence of loose bodies only (i.e., loose bodies with resolution of synovitis)

Simultaneous occurrence of CPPD and SC is rarely reported in the TMJ [3, 41, 42]. Herein, we present this unusual TMJ comorbidity in a 61-year-old Japanese man, delineate the molecular etiopathology of these diseases, reiterate the importance of confirmatory testing in minimizing diagnostic limitations and discuss surgery with the removal of lesions and synovectomy as the preferred choice of treatment. We present the case report following the CARE case report guidelines [43].

## Case presentation

The TMJ disorders were assessed following validated diagnostic criteria for temporomandibular disorders (DC/TMD) protocol [44].

### Patient information

A 61-year-old Japanese man was referred to our oral and maxillofacial surgery department in October 2020, with a chief complaint of persistent preauricular pain in the right cheek and restricted mouth opening for over 6 months. The patient was referred by a primary physician a month earlier for further investigations and surgical management when the frequency and intensity of pain increased and no longer responded to pharmacological treatment; mainly nonsteroidal anti-inflammatory drugs (NSAIDs). The patient had a history of clenching and grinding of teeth and medical history of controlled hypertension and type 2 diabetes mellitus (DM) by his family physician for many years. Hypertension was controlled at daily self-automated blood pressure (BP) measurements of systolic BP less than 130 mmHg and diastolic BP less than 80 mmHg, on oral amlodipine besylate 10 mg once daily. DM was controlled at hemoglobin A1c measurement values of 40–51 mmol/mol (5.8–6.8%), by oral administration of 5 mg linagliptin once a day after taking breakfast. The patient’s previous medical records also indicated that renal and hepatic function measurements were within normal limits, with nonremarkable history of any familial predisposition and surgical or traumatic injuries.

**Fig. 1:**
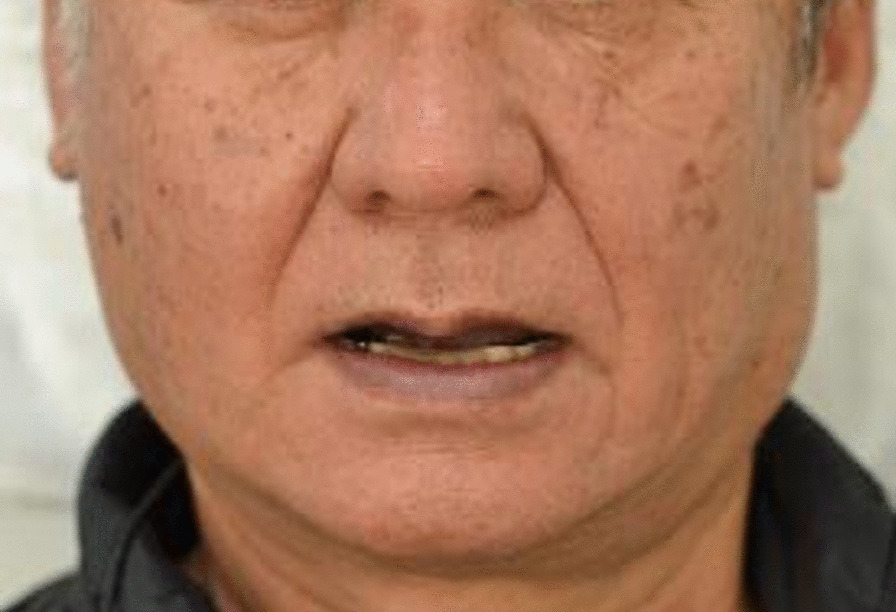
Frontal photograph. A 61-year-old Japanese man suffering from TMJ monoarthritis of the right side, showed a slight facial asymmetry and maximal mouth opening of approximately 15 mm

**Fig. 2:**
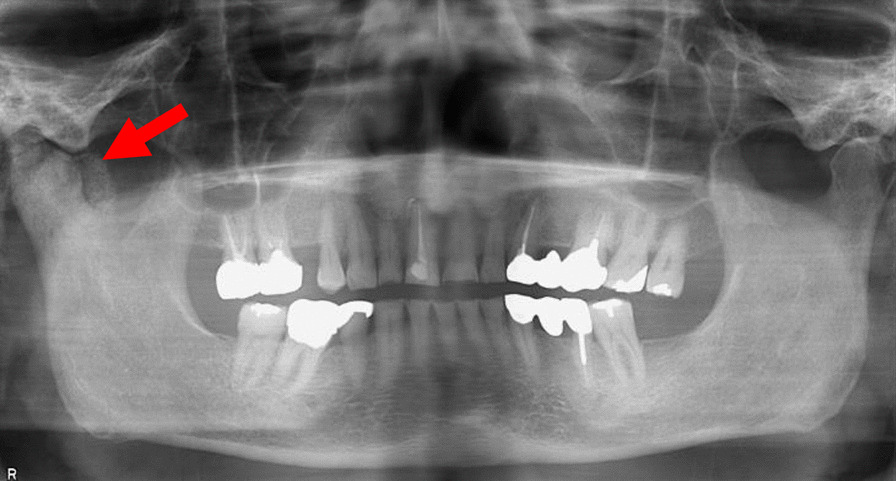
Panoramic radiograph. The red arrow indicates a large mass with a gravel-like appearance and calcified foci around the mandibular condyle with medial extension into the infratemporal fossa of the Right TMJ. In addition, occlusion features are observed including the mandibular first molars and canine teeth anteriorly in relation to the maxillary counterpart with incisional teeth wear for both upper and lower jaws

On examination, the patient was well-built, stood 178 cm, weighed 78 kg, and was in good general condition with no: pallor, edema, cyanosis, jaundice, cervical, or generalized lymphadenopathy. Systemic examinations were within normal limits. However, local examination revealed facial asymmetrical with mild swelling at the right preauricular region and limited mouth opening (mandibular active range of motion; AROM) of 15 mm, accompanied by replicated arthralgia involving masticatory muscles (myofascial pain with referral) at the visual analog scale of 8–10 in the right TMJ region (Fig. 1). The patient exhibited dysphonia and tenderness of the right TMJ. Further, excessive malocclusion (class III) and incisal teeth wear of the upper and lower jaws were observed (Fig. 2). There was no palpable mass or any evidence of infection observed around the right TMJ intraorally.

### Investigations

**Fig. 3:**
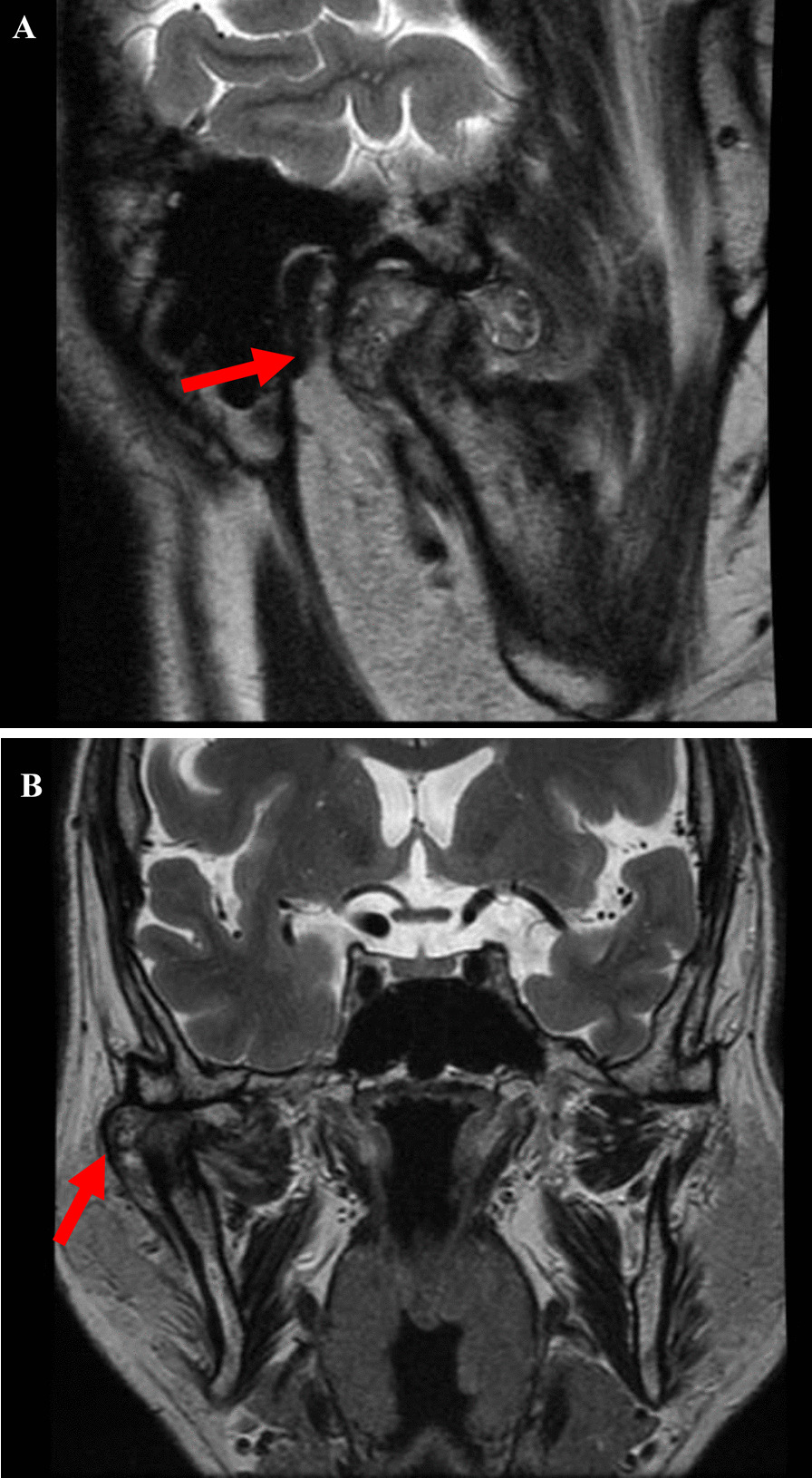
Preoperative MRI images. **A** Sagittal T1WI of the Right TMJ shows a fluid-filled mass expanding the joint space and multiple loose bodies. **B** Coronal PDWI showing heterogeneous signal intensity tissue mass within the capsule of the right TMJ between the right glenoid fossa and the mandibular condyle (red arrow)

**Fig. 4:**
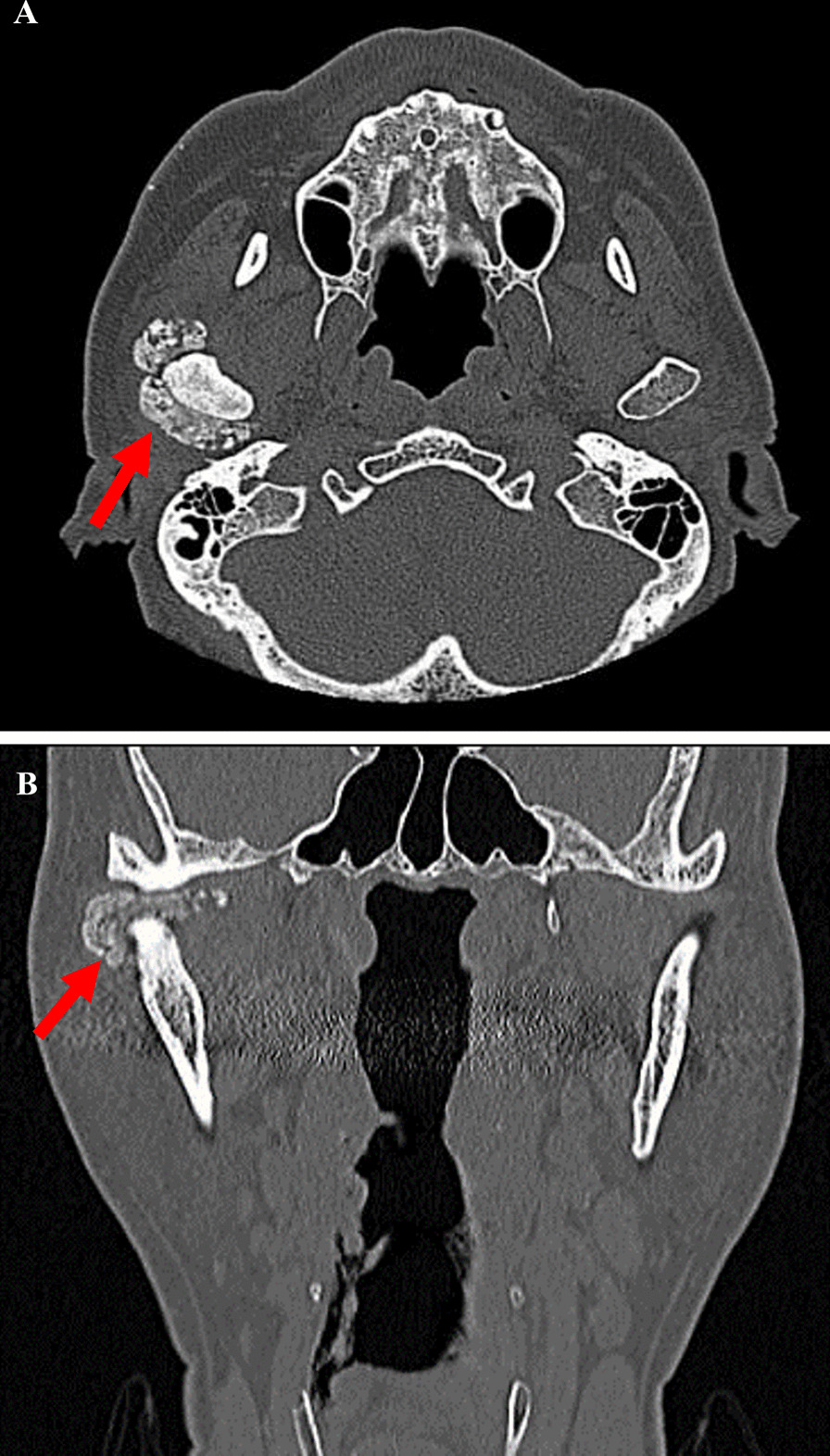
Preoperative CT images. **A** Axial CT image showing large intra-articular localized calcified lesions that abut the articular surface of the mandibular condyle of the Right TMJ. **B** Coronal CT image of the right TMJ indicated partial attachment of the calcified mass to the superior aspect of the mandibular condyle and cranial base

Radiography involved panoramic examination, contrast-enhanced computed tomography (CT), and magnetic resonance imaging (MRI) of the bilateral TMJ using protocols and recommendations highlighted in the recent literature [45]. Other investigations included routine hematological and confirmatory diagnostic histopathological evaluation. Panoramic radiography demonstrated the aforementioned malocclusion features and revealed horizontal bone resorption in the upper and lower jaws and extensive calcification-like opacities in the right TMJ (Fig. 2). Standard MRI protocols comprising proton density-weighted (PDWI) and T1-weighted (T1WI) sequences demonstrated dilation of the upper and lower joint spaces were with differing hypo intensity, no observable disc dislocation in the right TMJ. In addition, the heterogeneous signal intensity was scattered around the mandibular condyle and fossa. The left TMJ showed no abnormal findings (Fig. 3A, B). CT indicated a 38 mm-sized area of hard tissue, multiple rice-grain opacities (calcified loose bodies) around the right mandibular condyle, and osteosclerosis of the right articular head with no observable bone resorption. In addition, no tumorous lesions were identified in the contralateral TMJ (Fig. 4A, B). Hematological findings including uric acid levels were within normal ranges.

### Confirmation of diagnosis, treatment, and follow-up

**Fig. 5:**
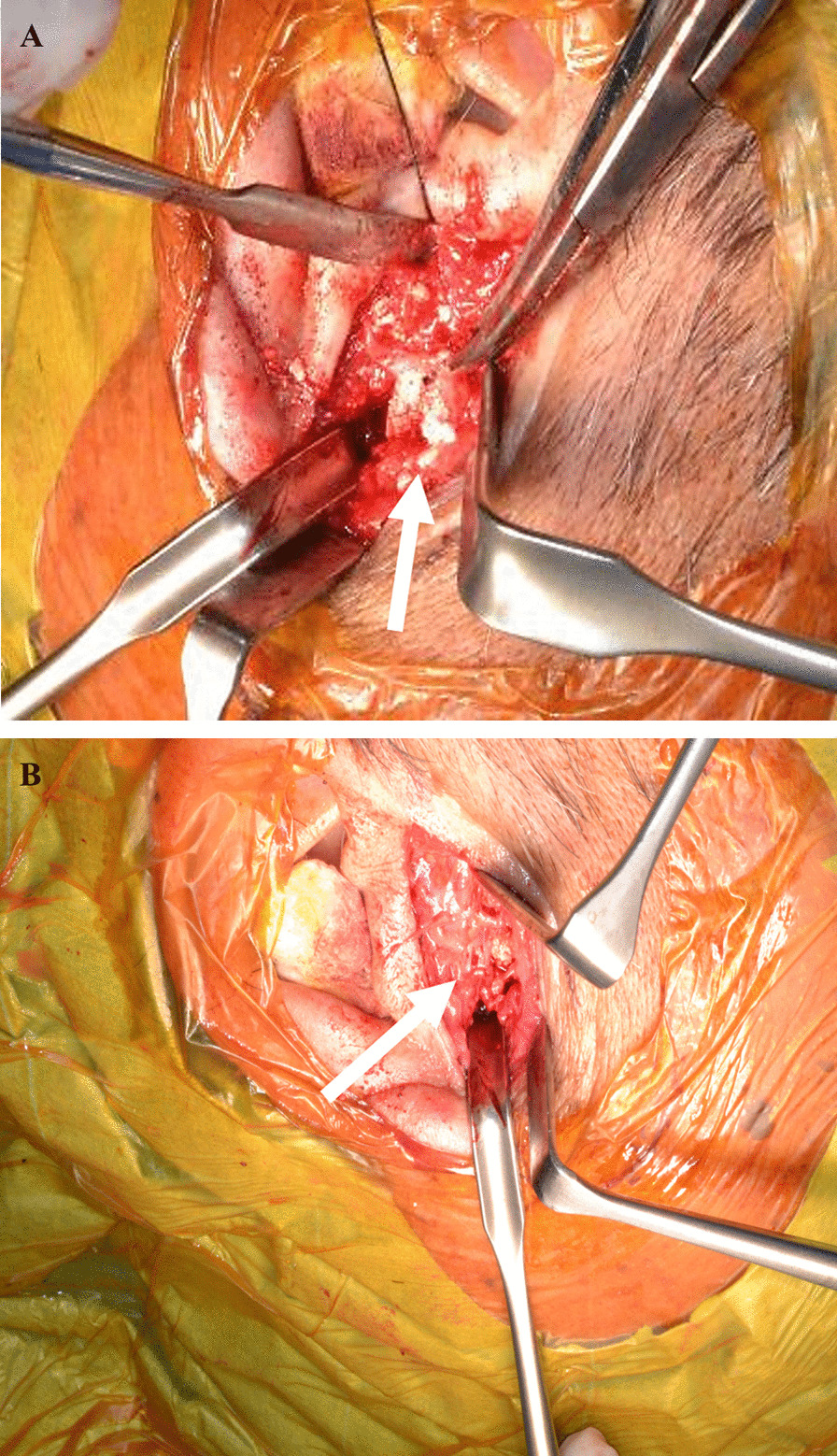
Intra-operative photographs. **A** Right preauricular surgical opening showing a tumor-like lesion of white substance in the infratemporal fossa (white arrow). **B** White arrow shows that the whitish calcified masses and loose bodies were removed

**Fig. 6:**
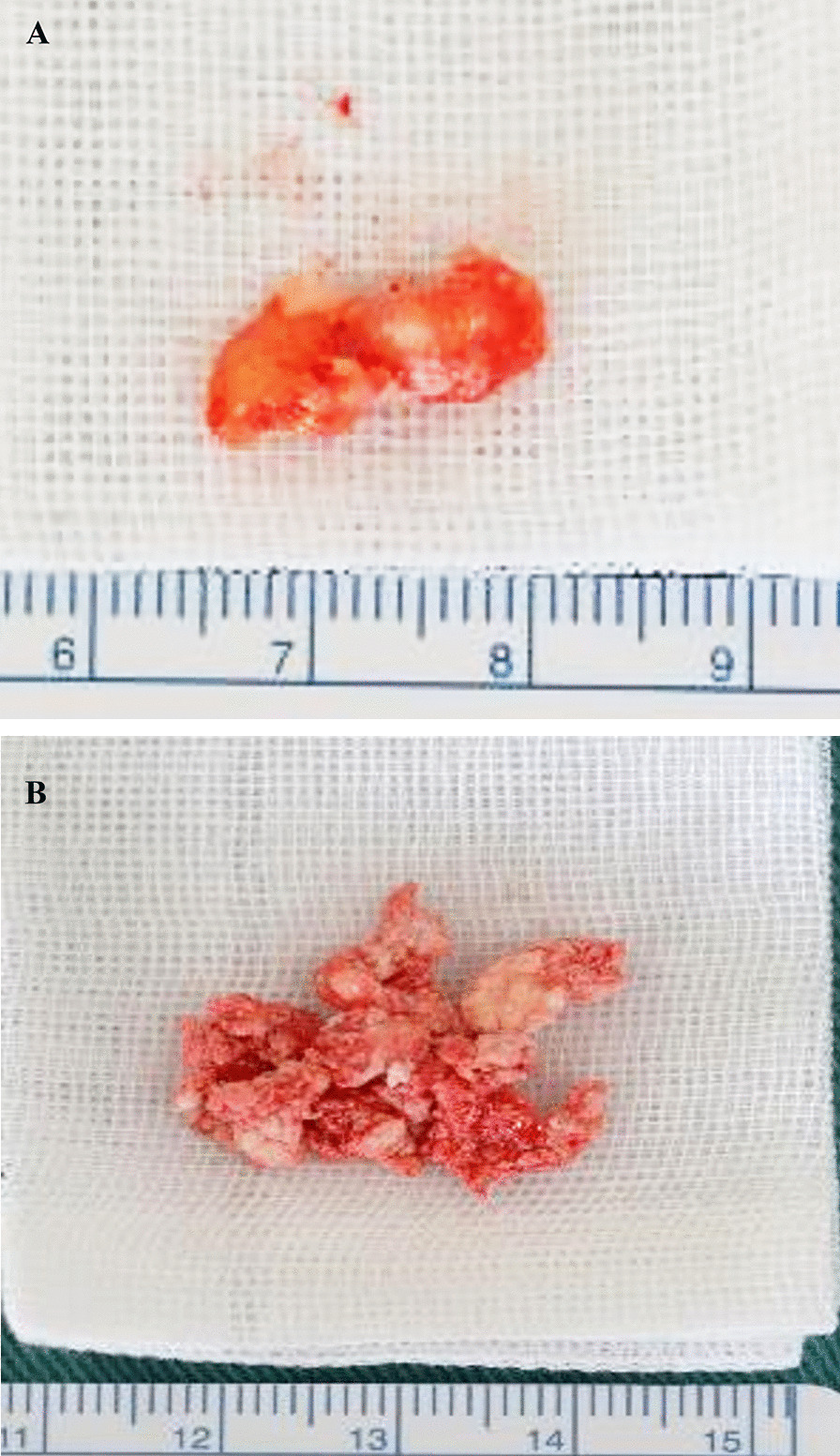
Photomicrograph of the excised masses. **A** Myxomatous cartilage-like structures of mixed sizes within a hyperplastic fibrous tissue. **B** Bone and chalk-like tissue specimen

**Fig. 7:**
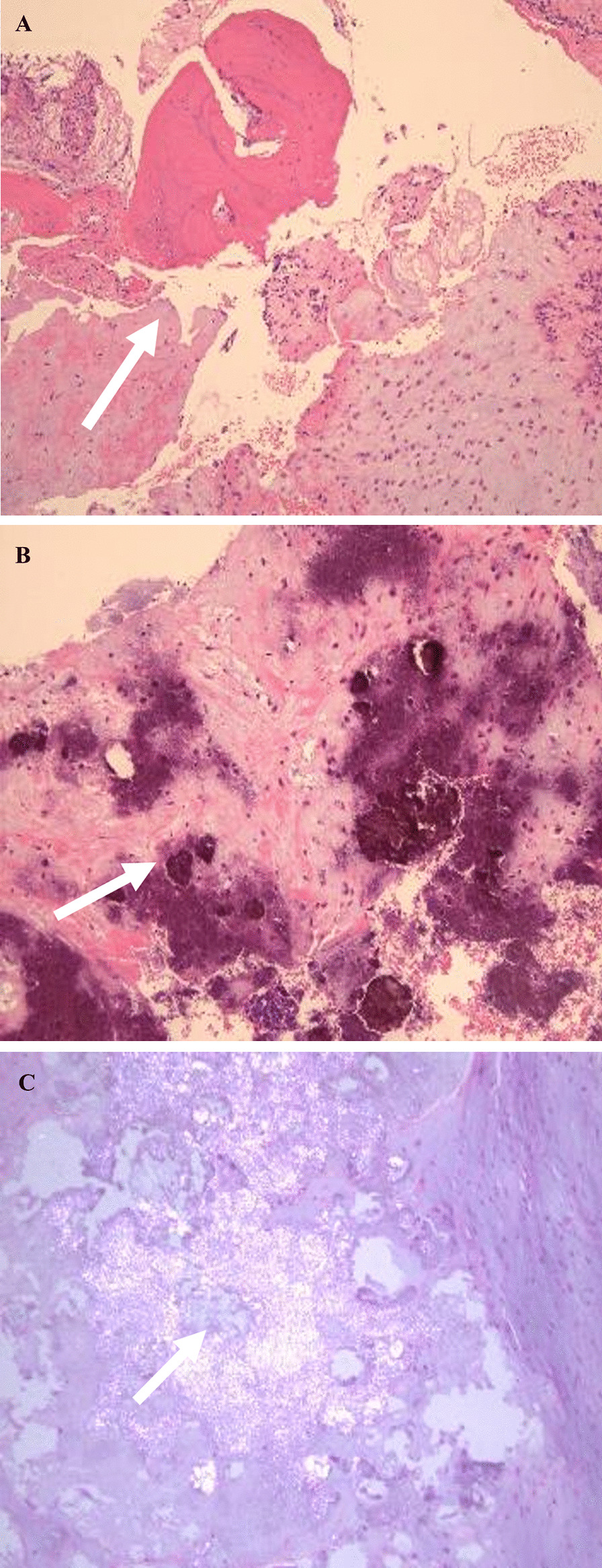
Histopathological examination of TMJ soft tissue obtained by surgical biopsy. **A** Hematoxylin and eosin staining section showing that the tumor-like mass was formed by aggregates of basophilic crystal deposits surrounded by a giant cell reaction (white arrow). **B** Hematoxylin and eosin staining show deposits of rhomboid-shaped crystalline material (white arrow). **C** Polarized microscopy shows positively birefringent rhomboid-shaped crystals (white arrow)

Considering the patient’s cardinal clinical features and radiological findings, the differential diagnosis of a tumor or tumor-like diseases such as pigmented villonodular synovitis (PVNS), CS, and a possibility of malignant chondrosarcoma of the right TMJ were made. Under general anesthesia, an open biopsy was performed to rule out malignancy and obtain a definitive diagnosis. Suffice it to say that, preparations for excisional biopsy amid overt features of malignancy were made. Briefly, surgery was approached through a right-sided preauricular incision, the shallow temporal artery rly identified and dissected, and the superior articular space was opened, which revealed a mass covered with a fibrous capsule. The mass had numerous white, cartilage-like hard tissues and chalk-like soft tissues of mixed sizes (Fig. 5A, B). The mass, which was surrounding the mandibular condyle, was bluntly removed using a mucous membrane exfoliator. No adhesions were observed between the mandibular head and the mass (Fig. 5). The mass was found exclusively in the superior joint space without much involvement of the condyle or the inferior joint space. No destructive change was observed on the surfaces of the condyle and mandibular fossa of the temporal bone. The excised myxomatous biopsy specimen comprising small and irregular cartilage-like structures within a hyperplastic fibrous tissue, forming numerous peripherally calcified or bone-like nodules as well as the chalk-like material (Fig. 6A, B), were sent to the pathology department for histopathological analysis, the surgical wound was cleaned, and the fascia and surrounding tissues were sutured with absorbable thread for closure. Histopathological evaluation of the open biopsy by hematoxylin and eosin staining revealed crystalline calcium deposits surrounded by fibroblasts, macrophages, foreign body-type giant cells, and granulomatous tissue of the hyperplastic synovial membrane containing crystal-like decalcified regions (Fig. 7A, B). Polarized light microscopy also showed the classical positive birefringent rhomboid, rhomboidal, and rod-like crystal deposits (Fig. 7C). No evidence of malignancy was observed. No further diagnostic approaches such as chemical analyses, scanning electronic microscopy/energy-dispersive x-ray spectroscopy, x-ray diffraction analysis (XRD), and inductively coupled plasma atomic emission spectroscopy were performed.

**Fig. 8:**
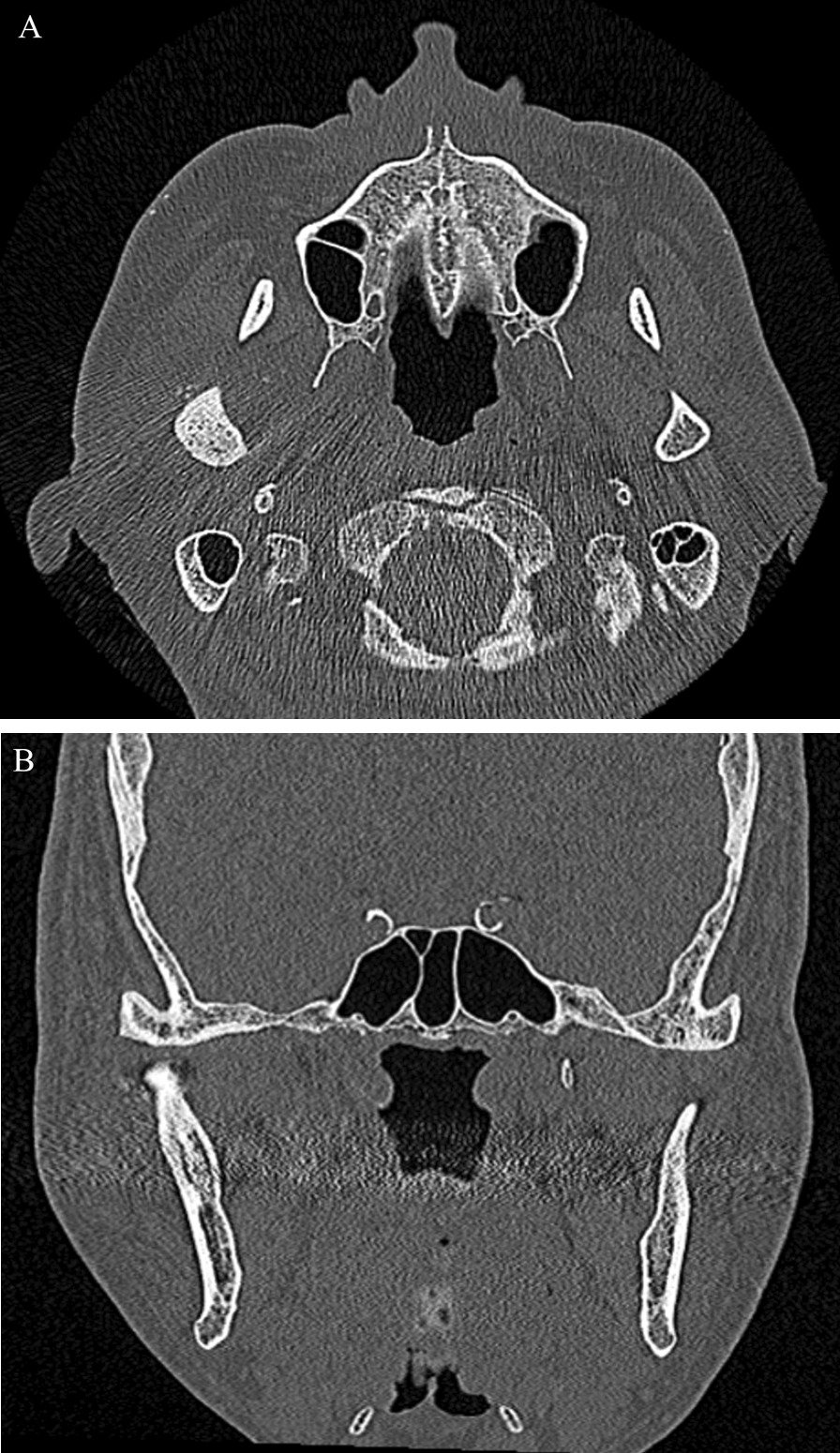
Postoperative CT images. **A** Axial CT images, and **B** coronal CT images confirmed the removal of the masses from the right TMJ

Considering all these findings, a pathological diagnosis of coexisting SC and CPPD in the right TMJ was made. In early January 2021, second-stage surgery was performed under general anesthesia to completely remove all the lesions, including synovectomy of the TMJ to prevent recurrence and improve mandibular function. Postoperatively, a mild motor disturbance was observed in the temporal branch of the right facial nerve, but the patient gradually recovered and was discharged. A 6 months post-surgery review indicated that although there was mild replicable pain in the right TMJ when opening the mouth, AROM had improved to 43 mm. Moreover, no obvious mandibular deviation, suggesting the possibility of a transient TMD-associated malocclusion, and no recurrence of symptoms was observed after nearly 1.5 years postoperatively (Fig. 8A, B).

### Patient perspective

**Fig. 9:**
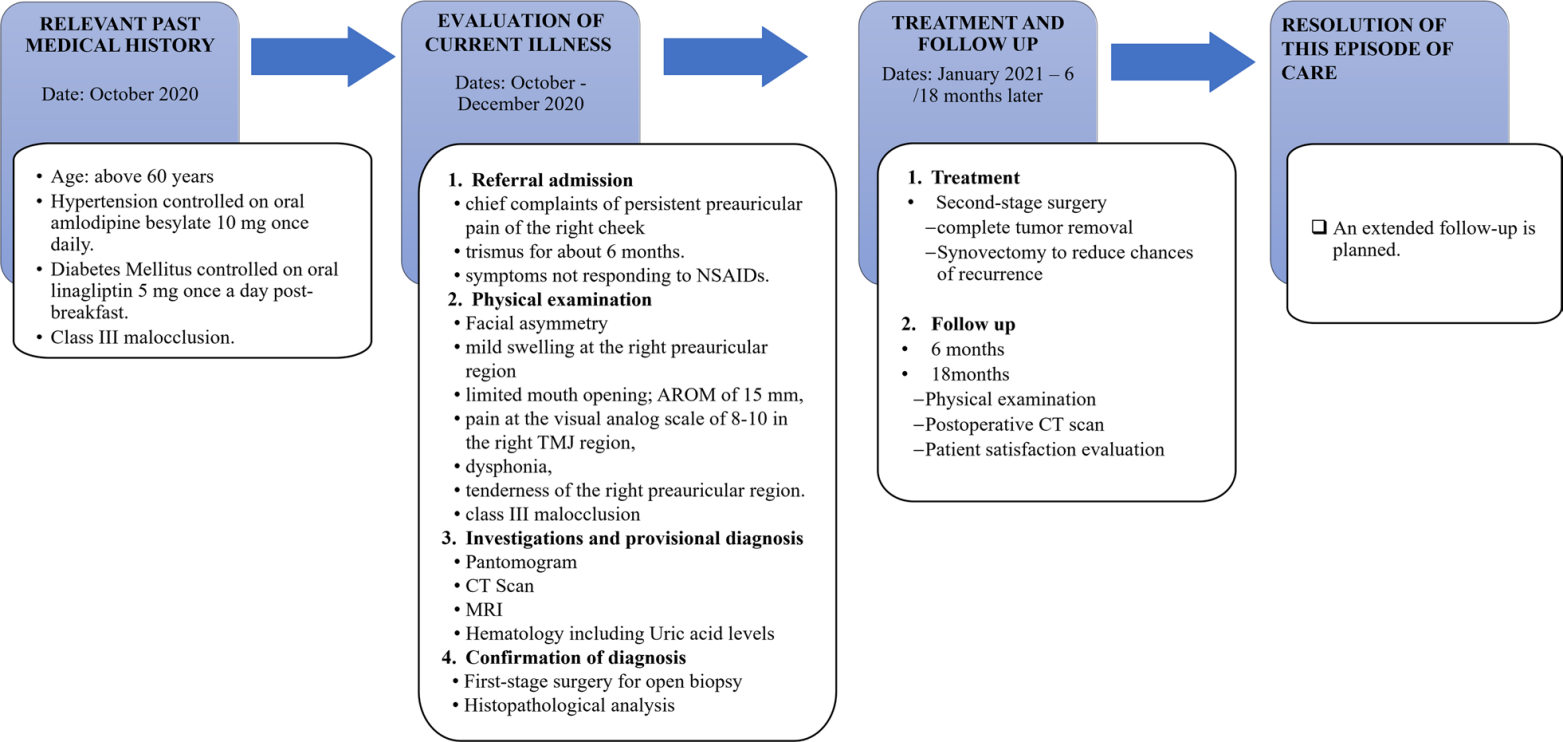
Historical and current information from this episode of care organized as a timeline

Due to the likelihood of a persistent malocclusion albeit of reduced severity, and recurrence of the TMD manifestations, an extended follow-up including a validated DC/TMD assessment is planned. Notwithstanding, the patient was very satisfied with the treatment outcomes and was very grateful to the entire medical team (Fig. 9).

## Discussion and conclusions

TMJ is indispensable to key facial activities such as jaw mobility, mastication, and verbal and emotional expression. CPPD and CS are among inflammatory arthropathies and neoplasm pathologies respectively, which commonly contribute to the annual incidence (4%) and prevalence (5–31%) of chronic TMJ pain, which is an economic burden to society [46, 47]. CPPD of the TMJ is generally rare, with only a few case reports published in the literature due to the associated difficulties in diagnosis [23, 24]. In contrast, although SC of the TMJ was previously considered a rare condition, the recent technological advancement in radiology dramatically minimized the diagnostic challenges and improved publication [22]. However, the coexistence of both CPPD and SC in the TMJ remains a rare condition [48].

A definitive diagnosis of CPPD is based on the presence of criteria I–IIb and considered likely with IIa–IIIb, as shown in Table 1. In the present case, although further diagnostic approaches, such as XRD and chemical analysis were not conducted, the patient had a great predisposition to DM, and concomitant clinical features such as chronic TMJ pain, in addition to the crystal deposition of previously described appearance [1–4], that displayed positive birefringence in polarized light (IIa). Moreover, CT, the best imaging modality for the disease revealed a typical calcification image (IIb), thus confirming the diagnosis of CPPD. Advanced imaging modalities, such as CT and MRI are very useful in making a reliable diagnosis and treatment plan for SC based on the criteria previously described [22, 36–38] and the histopathological classification shown in Table 2. In the present case, the patient had no history of trauma before the onset of illness, and radiology revealed joint effusion with multiple calcified loose bodies in a tumor-like lesion of approximately 38 mm with a well-defined border surrounding the mandibular condyle of the right TMJ. Moreover, histopathology confirmed the presence of only loose bodies, suggesting SC of phase III classification (Table 2). No evidence of malignancy or chondrosarcoma was observed in the patient and the findings of calcified loose bodies ruled out PVNS.

Therefore, taken together, the aforementioned findings suggested a coexistence of CPPD and SC in the right TMJ in the present case, the rare pathology. The mechanism by which SC and CPPD coexist is not well understood [3, 41, 42]. CPPD, a noninfectious inflammatory arthropathy could result from joint overloading, trauma, comorbidities such as metabolic disorders, osteoarthritis, and other TMJ disorders and later mediates SC [46]. On the other hand, primary SC characterized by chondrocyte differentiation of synovial pluripotent stem cells, and chondroid tissue production of unknown cause may simply occur in tandem with a familial CPPD [10]. The present case had a possibility of DM comorbidity and class III malocclusion, attributable to either primary SC or bruxism-induced stress, leading to the development of CPPD.

A recent report by Stack and Geraldine [49] well summarized and discussed the treatment options for CPPD. There currently are no CPPD- modifying therapies that can reduce articular calcification. Available pharmacological treatments aim to mitigate inflammation and symptoms' frequency and severity. The therapies include non-steroidal anti-inflammatory drugs (NSAIDs), colchicine, and corticosteroids that reduce the symptoms of CPPD. Anakinra and tocilizumab are recommended as a treatment for severe, refractory CPPD by EULAR. While crystal-targeted treatments such as nucleoside analogues and phosphocitrate are still under study but promising in attenuating calcification of human cartilage. Primary SC is treated with surgery including open or arthroscopic synovectomy and loose body resection due to severe symptoms that impact AROM [50]. Additional joint reconstruction or arthroplasty is recommended to prevent re-occurrence and degenerative joint disease. Secondary SC is treated with NSAIDs, and when symptoms persist or worsen, surgery is indicated.

The coexistence of both SC and CPPD is managed through excision of the lesion, anti-inflammatory therapy with NSAIDs, cleansing therapy, and follow-up. Excision of the loose bodies and pathological synovium is necessary, and some reports suggest that mandibulectomy is required if the lesion is large and invasive. However, Blankestijn et al. [51] reported that lesions rarely involve the mandibular head and do not necessarily require a mandibulectomy. Milgram [31, 40] also reported that synovectomy is not necessary for the final part of the third stage of the histopathological classification of SC. However, in the present case, surgery involved the complete removal of the loose bodies, CPPD lesions, and synovectomy to minimize the risk of recurrence. In addition, the arguable causal relationship between CPPD and SC of the TMJ suggested pathologically predisposed synovium and therefore, an indication for removal. The patient had been followed up for 1.5 years after surgery, without any post-surgery sequelae observed. However, because of the likelihood of persistent malocclusion and previously reported TMD recurrences [52, 53], an extended follow-up including a validated DC/TMD assessment of the patient has been planned.

In summary, although isolated cases of CPPD and SC of the TMJ are more prevalent in the literature, a monoarticular coexistence of these diseases is rare. This is often attributed to factors such as difficulties in diagnosing TMJ diseases, despite the advances in imaging technology and the reported interest in research and application of several different diagnostic approaches. Moreover, optimal treatment depends on several considerations. The present case delineated the molecular etiopathology of CPPD and SC and unveiled the need for continued deciphering mechanisms leading to the diseases' coexistence in the TMJ. In addition, the importance of confirmatory testing for accurate diagnosis was recapitulated and, in our opinion, appropriate management of the case was considered.

## Data Availability

The datasets used and/or analyzed for the case report are available from the corresponding author upon reasonable request.

## Abbreviations

ANKH: Progressive ankylosis protein homolog human gene
AROM: Mandibular active range of motion
BP: Blood pressure
CCAL: Chondrocalcinosis with early-onset osteoarthritis
CPP: Calcium pyrophosphate dihydrate crystal deposition
CPPD: Calcium pyrophosphate dihydrate crystal deposition disease
CT: Computed tomography
DM: Diabetes mellitus
HLA: Human leukocyte antigen
MRI: Magnetic resonance imaging
NSAIDs: Nonsteroidal anti-inflammatory drugs
PVNS: Pigmented villonodular synovitis
PPi: Inorganic pyrophosphate
SC: Synovial chondromatosis
TMJ: Temporomandibular joint
XRD: X-ray diffraction analysis
